# L1 and L2 contributions to English reading in middle school struggling readers

**DOI:** 10.1017/S1366728926101333

**Published:** 2026-04-24

**Authors:** Paul T. Cirino, Kelly T. Macdonald, Anny Castilla-Earls, David J. Francis, Arturo E. Hernandez

**Affiliations:** 1 University of Houston, USA; 2 Psychology, University of Houston, Houston, USA

**Keywords:** English learners, bilingualism, language, struggling readers, reading

## Abstract

This study evaluated English and Spanish language proficiency, and balance among these proficiencies, in relation to reading achievement in a sample of 161 middle school current and former English learners known to be struggling readers. Students were administered English and Spanish language assessments and also reported on their language usage; English reading outcomes (word reading, reading fluency, reading comprehension) were also assessed. Findings support the role of English proficiency in all three reading outcomes in this population. However, Spanish language skills, or indices that reflected the relative balance of these proficiencies, were not uniquely predictive. The present study adds nuance to the current literature and offers considerations for future work.

## Highlights

1. We examined L1 (Spanish) and L2 (English) language to L2 reading in at-risk children.
2. Contributions were considered from the perspectives of proficiency, balance and usage.
3. The primary contributor to L2 reading was L2 language proficiency.
4. Ways that L1 proficiency, usage and balance may influence reading are discussed.

The proportion of the population that speaks more than one language (bilinguals) continues to increase (Dietrich & Hernandez, 2022; National Center for Educational Statistics, 2024; Ortman & Shin, 2011). For children in the USA, Spanish is the most common language besides English (Dietrich & Hernandez, 2022). The present study examines language proficiency and use, as well as their relative balance, in children. We focus on an at-risk sample of middle school students – all were current or former English learners (ELs) with a Spanish-English language background and were struggling readers. Beyond this group being understudied, the focus on language and literacy is novel and important for several additional reasons, including that: (a) reading is emphasized throughout schooling and is a key life skill; (b) it is well known that language is the foundation of reading; and (c) reading is malleable, as there exists a strong evidence base of effective reading interventions (Scammacca et al., 2016). The purpose of this study is to identify patterns in the variability of the bilingual experience, which might then be leveraged to improve reading outcomes. The focus on middle school is relevant because by this point, students have had longstanding exposure to both heritage and societal languages. Also, by middle school, reading is increasingly used to gain information in *other* educational domains (e.g., science, history). The focus on English reading outcomes is relevant as in the USA it is the dominant language of instruction in middle school, even for students with diverse language backgrounds.

A frequently studied, though controversial, question about bilingualism is whether it offers an advantage, primarily in regard to executive function (EF). Studies do find such effects (Carlson & Meltzoff, 2008; Morales, Gómez-Ariza, & Bajo, 2013), though several large and recent reviews conclude that most results are very small, variable, or not significant (e.g., Gunnerud, Ten Braak, Reikerås, Donolato, & Melby-Lervåg, 2020; Lowe, Cho, Goldsmith, & Morton, 2021). Thus, in the present work, we evaluate whether bilingualism is related instead to reading outcomes, which is plausible given that reading is a language-based skill. Given that balance and/or degree of bilingualism has been proposed as a source of heterogeneity in the bilingual advantage literature (Thomas-Sunesson, Hakuta, & Bialystok, 2018; Yow & Li, 2015), the present study evaluates language balance directly rather than considering bilingualism only as a category (i.e., bilingual or not). This is particularly relevant in a developmental context where individual differences in students’ proficiency in English and Spanish are likely.

*Balanced* bilingualism has different meanings and is operationalized in different ways. For example, balance is often contrasted with dominance (of one language over another), and both can be gauged either from a *proficiency* perspective (e.g., using norm-referenced measures of language) and/or in terms of language *usage* (e.g., ratings of preference and usage), at an absolute level but more commonly along a continuum (Thomas-Sunesson et al., 2018). Balance could be taken to mean that proficiency and usage are *strong* in both their first and second languages (L1 and L2), though balance may also occur at weaker levels as well. The rationale for balance being important is that with greater balance comes more frequent switching and inhibition between languages relative to cases of clear dominance; there is empirical evidence to this point (Bialystok & Barac, 2012; Verreyt, Woumans, Vandelanotte, Szmalec, & Duyck, 2016). However, it is still the case that knowledge regarding bilingualism and its potential advantages comes mainly from studies of adults whose abilities in both L1 and L2 are well developed. Studies of children in general, and those at risk in particular, are less common, but bilingualism may also confer advantages even in such a context (White & Greenfield, 2017, in Head Start preschoolers). In this study, we consider both L1 and L2, their individual proficiencies and their balance, in terms of both proficiency and usage.

## 1. English learners, L1/L2 language and literacy

In the USA, 10% of students are categorized by their schools as ELs (Kena et al., 2015), a broad term which refers to students who also speak a language other than English. Depending on the locale, the term can be synonymous with low English proficiency (Genesee, Lindholm-Leary, Saunders, & Christian, 2005) or separated from it. Most ELs in the USA have Spanish as their first language (Hindman & Wasik, 2015; Passel, Cohn, & Lopez, 2011). ELs are at higher risk of academic difficulties than monolingual students (Francis, Rivera, Lesaux, Kieffer, & Rivera, 2006; Hemphill & Vanneman, 2011).

It is well known that language is crucial for reading. For example, the Simple View of Reading posits that reading achievement is influenced by a combination of decoding skill (single word reading) and oral language comprehension (Gough & Tunmer, 1986). Oral language skills play an increasingly stronger role in reading comprehension as students get older (Catts, Hogan, Adlof, & Barth, 2003; Foorman, Wu, Quinn, & Petscher, 2020). There is support for the Simple View across different orthographies (Koh & Joshi, 2023) and in Spanish-speaking ELs (Farnia & Geva, 2013; Geva & Massey-Garrison, 2013; Proctor, August, Carlo, & Snow, 2006), including being used as a frame for understanding reading of ELs in middle school (Mancilla-Martinez, Kieffer, Biancarosa, Christodoulou, & Snow, 2011) and older (Sparks & Patton, 2016) students.

There is large heterogeneity among ELs (August & Shanahan, 2017; Francis et al., 2019), including that related to age, language of instruction and sociodemographic variables (Hammer et al., 2014). In the present study, we focus specifically on the heterogeneity of language proficiency and use, given that our outcome is reading and language is the dominant predictor of reading skill. The idea that individual differences in L1 and L2 are important is reflected in bilingual theories of cross-linguistic transfer (Chen, Xu, Nguyen, Hong, & Wang, 2010; Genesee, Geva, Dressler, & Kamil, 2006; Kittle, Amendum, & Budde, 2024) and of interdependence (Cummins, 1981; Granados, Lorenzo-Espejo, & Lorenzo, 2022). Focusing on language and literacy in ELs who are struggling readers is particularly important, as they are the group most in need of reading assistance. Heterogeneity within this group could have implications for approaches to reading interventions for these students, which are less needed in cases where ELs are strong readers.

There is a relatively small but growing corpus of studies examining the heterogeneity of L1 and L2 language, and these fall into three interrelated groups: those that have formed latent profiles using both Spanish and English language measures (without predicting reading), those that predict reading with English and/or Spanish language measures (but without the use of latent profiles) and those that use latent profile membership to predict reading. These groups of studies are reviewed below.

### 1.1. Latent bilingual profiles

Swanson, Kudo, and Guzman-Orth (2016) and Swanson, Arismendi, and Li (2021) identified four latent groups in elementary students: two had average achievement (one with balanced language and one with unbalanced language) a third group was an English-dominant group and a fourth group was at risk of learning disabilities. This last group had lower scores on most measures of language, reading and math, as well as attention.

Macdonald et al. (2022) obtained three latent profiles of ELs in middle school using similarities and differences in L1 and L2 proficiency levels. Profile 1 was characterized by a balance between L1 and L2 and relatively high levels of language proficiency in each language (although still in the low average range) compared to other students. Profile 2 was characterized by a moderate imbalance, with Spanish levels *higher* than English. Finally, Profile 3 had a strong imbalance, with Spanish levels far *lower* than English levels. There was convergence between this person-centered approach with both a variable-centered approach (two factors, one each of English and Spanish), as well as with the use of a single continuous index of proficiency *and* balance (Vaughn & Hernandez, 2018). Finally, a self-report measure of language usage correlated with both English (*r =* .24) and Spanish (*r =* .56) proficiency, though differentially, and this helped distinguish the third profile from the others. Although that study helped establish relations among proficiency, balance, and usage, it did not do so from the perspective of reading, nor did it examine reading. Thus, a key goal of the present study was to examine the language proficiency factors and latent profiles (obtained from Macdonald et al., 2022) and relate these to English reading performances explicitly.

### 1.2. Reading prediction without bilingual profiles

Many studies focus on ELs in middle school and predict reading, but do not use latent profiles. Sometimes these studies include only English language measures (e.g., Nakamoto, Lindsey, & Manis, 2007). Others include both English and Spanish language predictors; these studies show mixed results regarding the contribution of L1 predictors of L2 reading (Huang, Bedore, Ramirez, & Wicha, 2022; Language and Reading Research Consortium [LARRC], Mesa, & Yeomans-Maldonado, 2019; Mancilla-Martinez & Lesaux, 2010; Marks et al., 2022; Miller et al., 2006; Nakamoto et al., 2008; Proctor, Harring, & Silverman, 2017). For example, Miller et al. (2006) found that Spanish oral language (semantic, syntactic, fluency, and discourse) predicted English reading scores beyond English language measures in a large sample of kindergarten to third-grade Spanish-English bilinguals; effects were stronger for English reading comprehension (English language skills added 23% over grade, Spanish language, 2%) than for English word reading efficiency (English language skills added 8%, Spanish language, 1%). Proctor et al. (2017), with students in second through fifth grade, found that Spanish syntax predicted fifth-grade English oral language skills and English reading comprehension, although English language controls were not included in the prediction of reading. In contrast to those studies, Nakamoto et al. (2008) found that the contributions of English decoding and oral language supplanted those of Spanish decoding and oral language in the prediction of English reading skills at sixth grade. Similarly, Mancilla-Martinez and Lesaux (2010) followed a sample of 173 students into fifth grade; English decoding and vocabulary predicted English reading comprehension, but analogous Spanish measures did not. Melby-Lervåg and Lervåg (2011) in a meta-analysis found a strong correlation (*r* = .44) of L1 phonological awareness to L2 decoding, but only a small relation (*r* = .04) between L1 oral language and L2 reading comprehension.

Group studies address a related issue, though in a different, more indirect manner. For example, Bialystok, Luk, and Kwan (2005) compared four groups of first-graders (one monolingual group and three bilingual groups of different language backgrounds including Spanish-English speakers) on decoding and phonological awareness tasks. They found stronger outcomes for all three bilingual groups compared to the monolingual group, especially for bilinguals who spoke two alphabetic languages. However, this study did not consider the role of balance between L1 and L2 or compare the contributions of English and Spanish to their preliteracy skills.

### 1.3. Reading prediction with bilingual profiles

We are aware of only two studies that considered latent language profiles of Spanish-English bilingual students with regard to how these profiles relate to reading; these are the studies most directly relevant to the present study. Grimm, Solari, Gerber, Nylund-Gibson and Swanson (2019) conducted a latent profile analysis with second-graders using measures of word reading, expressive language and receptive language in both languages. Three profiles emerged: a balanced profile and two unbalanced profiles, both of which had English skills in the average range but were differentiated by Spanish proficiency (either average or below average). Students with a balanced profile outperformed students with unbalanced profiles with regard to reading comprehension. This type of study suggests that balance affords additional benefit; it may be that, at least in the case of adequate English language skills, stronger (versus weaker) Spanish language skills indicate stronger overall language, thus affording some additional advantage toward reading comprehension.

In a sample of Spanish-English speaking preschoolers, Lonigan, Goodrich, and Farver (2018) used four objective measures of language to create subgroups of students characterized by differences in L1 and L2 proficiency as well as balance, via latent profile analysis. Their latent profiles demonstrated different patterns of performance across preliteracy assessments, but the dominant finding was that stronger English language abilities were associated with better English preliteracy skills. This study was with young students and also did not consider a range of reading outcomes.

## 2. Current study

Taken together, the above studies suggest that language-related variables (L1 and L2 proficiency), as well as the *balance* between them (as assessed via previously identified latent profiles), may contribute to reading outcomes, although the number of studies evaluating balance is small and the findings mixed, particularly with regard to balance. It is also rare in the literature to focus on middle school ELs who are struggling readers and to consider both variable-centered and person-centered approaches. Thus, the present study is both novel yet also builds upon existing work to further our understanding of L1 and L2 contributions to English reading skill.

## 3. Hypotheses

1. As reviewed above, prior findings for proficiency are mixed, but the preponderance of evidence suggests that English proficiency (relative to Spanish proficiency) would be more strongly related to English reading achievement.
2. We also expect that latent profiles of English and Spanish proficiency, and other metrics that explicitly reflect the *balance* among them, will relate to reading outcomes, over language proficiency per se (the Hypothesis 1 variables). Prior related studies have focused on reading comprehension, and so we expect these findings to be most prominent for this most complex form of reading, relative to word reading and fluency.
3. We expect that self-reported *language usage balance* will demonstrate an incremental contribution to reading outcomes over language proficiency per se.

## 4. Methods

### 4.1. Participants

The sample consisted of 161 sixth- and seventh-graders from public schools in the southwestern USA, all of whom were designated by their schools as ELs and as struggling readers on the basis of a statewide standardized reading test the prior year. Students came from six schools and 27 classrooms. The current sample is a subset of a larger parent study (*n* = 410) that included other measures and a reading intervention (Capin et al., 2024), and the overall focus of the parent study is on supporting ELs who are struggling readers. The present sample of 161 was a random selection (for feasibility and practicability reasons) of students enrolled in the larger study who were randomized to treatment or control and received language assessments in both languages. The current study utilizes only pretest data, so the effects of the intervention do not impact the results here. The sample is the same as that of Macdonald et al. (2022) though, as noted, reading was not examined in that study. All students were Hispanic, their home language was Spanish as reported by their schools and all were identified either as ELs or former ELs. Forty-eight percent of students were in the sixth grade and 41% were female. The mean age was 12.5 years (*SD* = 0.75 years). Seventeen percent of the sample had been previously identified by their school as requiring special education services, and 76% qualified for free/reduced lunch (and an additional 22% were missing these data). Other descriptive characteristics for this sample were not able to be obtained.

### 4.2. Procedures

All procedures were approved by the Institutional Review Boards of the investigators, including informed consent and assent from students. All assessments were administered by trained, supervised data collectors, including bilingual individuals.

### 4.3. Measures

Demographic information about age, gender, socioeconomic status and eligibility for special education services was obtained from schools. In addition, three types of measures were obtained from participants: language tests, self-report of language use and reading.

#### 4.3.1. Language proficiency measures

Nine measures of language proficiency (five in English, four in Spanish) were administered. *WJ-III Picture Vocabulary* (Woodcock, McGrew, Mather, & Shrank, 2007) assesses expressive semantics, requiring the student to provide a single word or phrase that matches pictured stimuli. *Woodcock-Muñoz Batería III Picture Vocabulary* (Batería III; Muñoz-Sandoval, Woodcock, McGrew, & Mather, 2005) subtest is the analogous task in Spanish. Psychometric properties in both English and Spanish are good, with test–retest reliabilities over .85 at this age. *Receptive One Word Picture Vocabulary Test* (ROWPVT-4; Martin & Brownell, 2011) assesses receptive semantic knowledge by evaluating a student’s ability to match a spoken word with an image of an object, action or concept. *ROWPVT-4, Spanish/Bilingual Edition* (Martin & Brownell, 2010), is a measure of *bilingual* receptive language, and thus, items are administered in Spanish and/or English; however, for the purposes of our study, we administered all items in Spanish (followed by English, if needed) in order to obtain an index of Spanish receptive vocabulary. Psychometric properties for the English and bilingual editions of the *ROWPVT* are good, with a test–retest reliability of 0.91 across all ages. *WJ-III Memory for Sentences* (Woodcock et al., 2007) evaluates expressive syntax and short-term auditory memory. Students are asked to remember and repeat single words, phrases and sentences presented orally, with items gradually increasing in their grammatical complexity. *Woodcock-Muñoz Batería III Memory for Sentences* (Muñoz-Sandoval et al., 2005) is the analogous task in Spanish, and both English and Spanish tasks have a median reliability of .89 at this age. The *Sentence Assembly* subtest from the Clinical Evaluation of Language Fundamentals Fourth Edition (CELF-4; Semel, Wiig, Secord, & Langdon, 2006) is an additional test of expressive syntax in English which assesses a student’s ability to formulate grammatically and semantically correct sentences following the visual and verbal presentation of words. The CELF-4 has good psychometric properties, with Cronbach’s alpha ranging from .70 to .91 across subtests. *WJ-III Understanding Directions* (Woodcock et al., 2007) is a measure of receptive syntax which asks the student to listen to a sequence of instructions and follow directions by pointing to various objects in a colored picture. *Woodcock-Muñoz Batería III Understanding Directions* (Muñoz-Sandoval et al., 2005) is the analogous task in Spanish. Psychometric properties in both English and Spanish are good, with a median reliability of .77 at this age. Reliabilities for these measures in this sample were adequate, ranging from .71 to .98.

#### 4.3.2. Self-report language usage

The *ROWPVT-4, Spanish/Bilingual Edition*, contains a self-report measure of language usage with a three-point Likert-type scale, where 1 = “Mostly Spanish,” 2 = “Half Spanish, Half English” and 3 = “Mostly English.” The items assess the individual’s language use across a range of contexts, including which language they use to speak to parents, siblings, peers and teachers, as well as which language they use to read, watch television, and so forth. Reliability for this measure was *α* = .67. We considered this measure categorically, by inspection of the distributions of responses, resulting in three groups: mostly English usage, mostly Spanish usage and balanced usage.

#### 4.3.3. Reading measures

Participants were administered common assessments of word reading accuracy, reading fluency and reading comprehension. Subtests from the *Kaufman Test of Educational Achievement Third Edition* (KTEA-3, Kaufman, 2014) were used, including *Letter and Word Recognition* and *Word Recognition Fluency.* Split-half reliability estimates for seventh-graders taking these subtests are .96 and .89, respectively (Kaufman, 2014). The *Gates MacGinite Reading Comprehension Test Revised* (GMTR; MacGinitie, MacGinitie, Maria, & Dreyer, 2000) was used to assess reading comprehension. Alternate form reliability for the GMTR ranges from .80 to .87 (MacGinitie et al., 2000). Age-based standard scores were used for the three reading outcomes in the analyses.

### 4.4. Analyses

Analyses focused on descriptive statistics, correlations and regression. Primary analyses were conducted in SAS, with the two language factor scores of Spanish and English proficiency exported from Mplus (these results were convergent with those conducted directly in Mplus). Latent profiles were exported from Mplus to SAS. Regression diagnostics demonstrated adequate normality, linearity, homogeneity of variance and independence of errors. There was collinearity for one portion of the steps of regression (described below). We evaluated four potential demographic covariates in terms of their relevance for reading outcomes: age, gender, free/reduced lunch, and special education eligibility (SPED). Of these, gender and free/reduced lunch status were not related to any reading outcome (all *p* > .05) and so were not further considered. Age was related to both word reading (*p <* .002) and fluency (*p <* .001), though not comprehension, and SPED was related to all three reading outcomes (all *p <* .001). Thus, for word reading and fluency, age and SPED were the final covariates. For reading comprehension, SPED was a covariate, and we included word reading here as well, given its known relation to (*p* < .001 here) and key role as a building block for reading comprehension.

Three sets of multilevel regression models were used to evaluate hypotheses, one for each reading outcome (word reading accuracy, oral reading fluency, reading comprehension), and squared semi-partial correlations were computed as estimates of effect size. Given that students were clustered in classrooms, intraclass correlations were computed, which were .02, .05 and .13, for word reading, reading fluency and reading comprehension, respectively. Regression models were run in PROC MIXED in order to isolate variability at the individual level by controlling for classroom-level variance; language variables were centered at the classroom means and included both the centered variables and the classroom means in the mixed models.

Models were built in steps for each of the three reading outcomes. Step 1 included only covariates. Step 2 added English and Spanish proficiency factor scores (classroom-centered scores and classroom means), which addressed variable-centered contributions of L1 and L2 (Hypothesis 1). Step 3 addressed balance (Hypothesis 2) by adding information to Step 2 models, in three different ways. First (Step 3a), a continuous metric was added that combined balance and proficiency. There was extreme collinearity for the balance metric when both English and Spanish proficiency variables (from Step 2) were also included (not surprising since this index includes both proficiency scores). But given that the goal of Step 3a was to examine the balance metric specifically, the measure of Spanish proficiency was excluded for this Step 3a only, which resolved the collinearity issue. Collinearity was not a concern for any other model-building step. Second (Step 3b), we added to Step 1 and Step 2 variables the person-centered latent profile membership as determined from Macdonald et al. (2022); specifically, profiles were dummy-coded with the balanced group serving as reference. Third and finally (Step 3c), addressing Hypothesis 3, self-reported language usage (also dummy-coded, with balanced usage as the reference group) was added to the Step 1 and Step 2 variables.

## 5. Results

Table 1 includes descriptive statistics and correlations between and among the three reading outcomes (sight word reading, reading fluency and reading comprehension) and language variables. Reading scores were all in the low average range, highlighting that the students here were struggling readers. Results from the three sets of regression analyses are presented in Tables 2–4, with Hypothesis 1 results appearing in the top portion of each table (Steps 1 and 2), Hypotheses 2 corresponding to Steps 3a and 3b in each table and Hypothesis 3 corresponding to Step 3c in each table.

**Table 1.** Descriptive statistics, reliabilities and correlations among language and reading variables

		1	2	3	4	5	6	7
1	Word reading	–						
2	Reading fluency	.73**	–					
3	Reading comprehension	.34**	.45**	–				
4	English proficiency	.35**	.35**	.43**	–			
5	Spanish proficiency	.13	.06	.16*	.04	–		
6	Continuous metric of Balance and proficiency	.30**	.23*	.39**	.67**	.71**	–	
7	Self-report language usage	.10	.15	.12	.24*	−.56**	−.23*	–
	*Mean*	85.32	82.35	80.51	0	0	0.06	2.17
	*SD*	13.89	12.33	9.64	0.83	0.94	1.01	0.35
	*Reliability*	.94	.93	.77				.67

*Note*: Language scores were below age expectation on average (mean English SS = 79, mean Spanish SS = 75). Mean scores are 0 for the English proficiency and Spanish proficiency factor scores.

*p < .05; ^**^p < .001.

**Table 2.** Regression results predicting word reading

	Fixed effect	Coefficient	*SE*	Semipartial *ω^2^*	*t*
Step 1:	Intercept	141.89	17.57		8.08***
Covariates	Age	−4.33	1.41	0.044	−3.08**
	SPED^+^	−12.28	2.62	0.109	−4.69***
Pseudo-*R* ^2^	0.22***				
Step 2:	Intercept	160.24	18.60		8.62***
Language proficiency	English (individual)	4.73	1.28	0.059	3.68***
	Spanish (individual)	0.61	1.31	−0.004	0.46
	English (classroom)	8.20	2.89	0.033	2.84**
	Spanish (classroom)	1.99	1.82	0.001	1.09
Pseudo-*R* ^2^	0.28***				
Step 3a:	Intercept	162.61	18.73		8.68***
Continuous	(individual)	0.18	1.52	−0.005	0.12
Balance metric	(classroom)	3.58	2.59	0.015	1.38
Pseudo-*R* ^2^	0.28***				
Step 3b:	Intercept	162.18	19.16		8.47***
Latent profiles^a^	Profile 2	−1.36	3.22	−0.004	−0.42
	Profile 3	−2.28	5.39	−0.004	−0.42
Pseudo-*R* ^2^	0.29***				
Step 3c:	Intercept	157.80	18.37		8.59***
Language usage^b^	English usage	1.48	2.67	−0.003	0.56
	Spanish usage	−4.16	2.65	0.007	−1.57
Pseudo-*R* ^2^	0.29***				

	Covariance parameter estimates			
	Intercept (classroom)		Residual	
	Estimate (*SE*)	*Z*	Estimate (*SE*)	*Z*
Step 1	4.12 (6.56)	0.63	153.82 (17.95)	8.57***
Step 2	0.82 (4.51)	0.18	138.03 (15.97)	8.64***
Step 3a^c^			138.48 (15.43)	8.97***
Step 3b	1.28 (4.81)	0.27	137.42 (15.95)	8.62***
Step 3c^c^			136.38 (15.20)	8.97***

*Note:* Parameter estimates include variables from prior Steps (Step 2 parameters are generated from a model that also includes Step 1 variables). Step 3 parameters (a, or b, or c) include variables from both Steps 1 and 2, with the exception of Step 3a, which excluded Spanish proficiency due to collinearity as described in text. For Step 3b, the three latent profiles were dummy-coded, with Profile 1 as the reference group. For Step 3c, usage is also dummy-coded, with balanced usage as the reference group.

*p < .05;**p < .01; ***p < .001.

+ SPED, special education eligibility status.

a Reference = Profile 1; ^b^Reference = balanced usage; ^c^Covariance parameter estimates not available for intercept because the estimated G matrix is not positive definite.

**Table 3.** Regression results predicting fluency

	Fixed effect	Coefficient	*SE*	Semipartial *ω^2^*	*t*
Step 1:	Intercept	154.64	16.88		9.16***
Covariates	Age	−5.56	1.36	0.081	−4.10***
	SPED^+^	−10.12	2.27	0.097	−4.46***
Pseudo-*R* ^2^	0.33***				
Step 2:	Intercept	173.22	16.56		10.46***
Language proficiency	English (individual)	3.73	1.09	0.047	3.43***
	Spanish (individual)	−0.78	1.11	−0.002	−0.70
	English (classroom)	10.41	2.58	0.067	4.03***
	Spanish (classroom)	2.02	1.61	0.003	1.26
Pseudo-*R* ^2^	0.36***				
Step 3a:	Intercept	175.20	16.59		10.56***
Continuous	(individual)	−1.83	1.28	0.004	−1.43
Balance metric	(classroom)	3.23	2.29	0.004	1.41
Pseudo-*R* ^2^	0.37***				
Step 3b:	Intercept	169.98	16.67		10.20***
Latent profiles^a^	Profile 2	2.13	2.74	−0.002	0.77
	Profile 3	−0.47	4.59	−0.004	−0.10
Pseudo-*R* ^2^	0.36***				
Step 3c:	Intercept	168.66	16.15		10.44***
Language usage^b^	English usage	3.20	2.28	0.004	1.40
	Spanish usage	−3.07	2.26	0.004	−1.36
Pseudo-*R* ^2^	0.36***				

	Covariance parameter estimates			
	Intercept (classroom)		Residual	
	Estimate (*SE*)	*Z*	Estimate (*SE*)	*Z*
Step 1	13.90 (10.69)	1.30	109.51 (13.35)	8.20***
Step 2	3.37 (5.23)	0.65	98.90 (11.74)	8.43***
Step 3a	2.78 (5.13)	0.54	98.20 (11.69)	8.40***
Step 3b	2.25 (4.98)	0.45	99.12 (11.80)	8.40***
Step 3c	1.66 (4.69)	0.35	97.84 (11.64)	8.41***

*Note:* See Table 2 note, which also applies here.

*p < .05;**p < .01; ***p < .001.

+ SPED, special education eligibility status.

a Reference = Profile 1.

b Reference = balanced usage.

**Table 4.** Regression results predicting comprehension

	Fixed effect	Coefficient	*SE*	Semipartial *ω^2^*	*t*
Step 1:	Intercept	69.16	4.77		14.51***
Covariates	SPED^+^	−6.32	1.91	0.052	−3.31**
	Word reading	0.15	0.05	0.038	2.86**
Pseudo-*R* ^2^	0.30***				
Step 2:	Intercept	72.28	4.57		15.80***
Language proficiency	English (individual)	2.90	0.92	0.041	3.16**
	Spanish (individual)	0.21	0.90	−0.004	0.24
	English (classroom)	7.56	1.78	0.078	4.24**
	Spanish (classroom)	1.15	1.18	0.000	0.98
Pseudo-*R* ^2^	0.31***				
Step 3a:	Intercept	71.78	4.54		15.80***
Continuous	(individual)	0.52	1.04	−0.003	0.50
Balance metric	(classroom)	2.50	1.62	0.006	1.54
Pseudo-*R* ^2^	0.30***				
Step 3b:	Intercept	70.25	4.85		14.50***
Latent profiles^a^	Profile 2	2.39	2.19	0.001	1.09
	Profile 3	2.30	3.70	−0.003	0.62
Pseudo-*R* ^2^	0.30***				
Step 3c:	Intercept	71.47	4.54		15.73***
Language usage^b^	English usage	5.30	1.80	0.034	2.95**
	Spanish usage	0.82	1.81	0.003	0.45
Pseudo-*R* ^2^	0.33***				

	Covariance parameter estimates			
	Intercept (classroom)		Residual	
	Estimate (*SE*)	*Z*	Estimate (*SE*)	*Z*
Step 1	7.76 (5.46)	1.42	69.11 (8.26)	8.37***
Step 2	0.64 (3.10)	0.21	64.53 (7.76)	8.32***
Step 3a^c^			64.63 (7.23)	8.94***
Step 3b^c^			64.69 (7.23)	8.94***
Step 3c*			61.78 (6.91)	8.94***

*Note:* See Table 2 note, which also applies here.

*p < .05;**p < .01; ***p < .001.

+ SPED = special education eligibility status.

a Reference = Profile 1.

b Reference = balanced usage.

c Covariance parameter estimates not available for intercept because the estimated G matrix is not positive definite.

### 5.1. The roles of English and Spanish proficiency in reading outcomes (Hypothesis 1)

For word reading, covariates accounted for 22% of the variance at Step 1. When English and Spanish factor scores were added in Step 2 (to the variables in Step 1), all predictors together accounted for 28% of the variance, with a significant unique contribution from English proficiency (9.2% of the unique variability in word reading), with 5.9% at the individual student level (*p <* .001) and 3.3% at the classroom level (*p* = .006). Spanish proficiency was not a unique predictor at the individual (*p =* .644) or classroom (*p =* .277) levels.

For reading fluency, covariates accounted for 33% of the variance at Step 1. In Step 2, adding English and Spanish proficiency scores to Step 1 variables accounted for 36% of the total variance in fluency, with again a unique contribution from English proficiency (11.4% variance; individual, 4.7%, and classroom, 6.7%; both *p* < .001). Again, Spanish proficiency did not contribute to fluency at the individual (*p* = .485) or classroom (*p =* .211) levels.

For reading comprehension, covariates accounted for 30% of the variability, with word reading accounting for 3.8% of the unique variance. In Step 2, 31% of the variability in comprehension was accounted for, with a significant contribution from English proficiency, which uniquely explained 11.9% of the variance (individual, 4.1%, *p =* .002; classroom, 7.8%, *p* < .001), even with the covariates of Step 1 present. As with the other reading outcomes, Spanish proficiency did not contribute to comprehension at the individual (*p* = .813) or classroom (*p =* .331) levels.

### 5.2. The incremental role of balance in reading outcomes (Hypothesis 2)

We next considered metrics that integrate both proficiency and balance for the three reading outcomes. For Step 3a, focused on the continuous single metric, this variable was added to Step 1 covariates and to English proficiency from Step 2, as described in the analysis plan. In this context, individual-level variability in the continuous metric did not significantly predict word reading (*p* = .905), fluency (*p* = .156) or comprehension (*p* = .620). Variability in the continuous metric at the classroom level was also not significantly predictive of any reading outcome (all *p* > .05).

In Step 3b, we considered how the latent profiles related to reading skills while also considering demographics (Step 1 variables) and language proficiency in English and Spanish (Step 2 variables). Whether latent profiles were treated deterministically or with the pseudo-random draws approach, profile membership did not significantly predict word reading, fluency or comprehension over and above language proficiency (all *p* > .05).

### 5.3. The incremental role of self-reported language usage in reading skills (Hypothesis 3)

In the final step (3c) of the regression models, the self-report metric was added to all Step 1 and Step 2 variables. For word reading and reading fluency, the dummy-coded self-report categories (English usage, Spanish usage), relative to balanced usage, were not uniquely significant (all *p* > .05). For reading comprehension, the usage variable was significant, with a higher level of English usage relative to balanced usage accounting for 3.4% unique variance (*p* = .004); however, a higher level of Spanish usage relative to balanced usage was not significantly related to comprehension (*p* = .651).

## 6. Discussion

The purpose of the present study was to evaluate the roles of L1 and L2 proficiency *as well as* balance in predicting L2 reading outcomes in a sample of middle school Spanish-English-speaking current and former ELs who were struggling readers. Results demonstrated significant contributions of L2 (English) proficiency, but not L1 (Spanish) proficiency, to all reading outcomes. Variables that integrate balance and proficiency were also not uniquely predictive.

### 6.1. The roles of English and Spanish language proficiency for reading outcomes

As reviewed in the Introduction, prior work is mixed with regard to the role of Spanish oral language skills for English reading outcomes (e.g., Miller et al., 2006; Nakamoto et al., 2008). Results of the present study, though, clearly indicate that in this sample, English oral language skills supersede contributions from Spanish oral language skills in the prediction of English word reading, fluency and comprehension. Importantly, both individual- and classroom-level variability accounted for unique variance in all three reading outcomes, meaning that L1 proficiency of students in the same classroom is related to individual variation in reading performance, particularly for more complex skills such as reading comprehension. Important differences in three specific sample characteristics (age, variability in instructional programming and level of Spanish proficiency) across studies likely impact variability in the results of prior studies and conclusions regarding the role of L1 language processes in L2 reading throughout development. Each is discussed in turn.

First, it may be that Spanish language individual differences have an impact on English reading skills, but only or primarily at younger ages. Most studies finding support for the role of Spanish language skills in English reading outcomes were conducted at the preschool, kindergarten or early elementary levels (i.e., Miller et al., 2006; Proctor et al., 2006, 2017), though some studies even at this younger age level did not find support for the role of Spanish skills in English preliteracy outcomes (e.g., Lonigan et al., 2018). Of course, it is unknown whether Spanish effects would have been evidenced in the present sample, had these students been tested several years prior. The present negative results do raise the possibility that these effects wash out by middle school. Conversely, as a Romance language, it is also possible that Spanish language skills may exert a greater influence on English development as the number of Latin-based words introduced to the English lexicon increases in adolescence and young adulthood (Hernandez et al., 2021). Longitudinal studies that follow students from young ages through elementary school and into middle/high school are needed to better understand the impact of development on the relationship between L1 language skills and L2 reading in a more direct manner. It should also be noted that the strongest effect from the Melby-Lervåg and Lervåg (2011) meta-analysis was for the contribution of L1 phonological awareness to L2 decoding, although Spanish phonological awareness was not assessed in the present study. Doing so, particularly in the context of a longitudinal design through middle school and beyond, would make for an additional interesting future study.

A second but related source of variability across studies is with regard to early instructional programming and specifically language of instruction, which may also have a larger impact at younger ages such as early elementary school. Many of the studies reporting a significant effect of Spanish oral language have been conducted with students who are Spanish language dominant and had received the majority of instruction in Spanish and who were gradually transitioning to English instruction (i.e., Miller et al., 2006; Proctor et al., 2006). In the context of strong Spanish language skills, students may be more likely to need to, and simultaneously be able to, harness those skills to support English reading development. In contrast, the middle school students in our sample were receiving all instruction in English and had *lower* Spanish skills on average relative to their English skills. Even where instructional curricula do have effects, these may dissipate over time. Nakamoto et al. (2008) for instance, found that in sixth grade, Spanish oral language did not predict English reading comprehension in a sample of bilinguals who had all participated in an early transitional bilingual curriculum starting in kindergarten and who had continued to receive some degree of instruction in Spanish throughout elementary school. Unfortunately, we did not have information about the early instructional programming of the students in this sample, though future work should attempt to incorporate this.

Third, it is also possible that a certain level of Spanish language skill, and/or a certain threshold of English decoding skill, is needed in order for Spanish language to bolster English reading comprehension (Chen et al., 2010). The students in this sample had overall low levels of Spanish language (see Macdonald et al., 2022) and English decoding (see Table 1), and only English language made a contribution to English reading; thus, if some threshold was relevant, it is likely many of the students here would have been below any such threshold. Further support for this possibility comes from studies that have found cross-linguistic interactions between Spanish oral language and English decoding (Nakamoto et al. 2008; Proctor et al., 2006); in these cases, Spanish skills contributed to English reading comprehension in strong decoders but not in weaker decoders. Though not the focus of our study since our aim was to better understand these relationships among ELs who are struggling readers, future work should replicate and extend our findings with middle school samples that have a greater representation of average or high Spanish proficiency as well as stronger English decoding. Overall, our finding that English language proficiency and English single word reading predicted English reading comprehension is consistent with well-researched theories of reading development, specifically the Simple View (Gough & Tunmer, 1986), providing novel support for this theory in the context of middle school Spanish-English-speaking bilinguals who are also struggling readers.

### 6.2. The incremental role of balance for reading outcomes

Once we positioned the roles of Spanish and English oral language in English reading, we then sought to evaluate the role of *balance* between Spanish and English proficiency for English reading outcomes. In constructing our hypotheses about the role of balance in reading, we followed the literature in taking a strengths-based perspective, which suggested that if there are advantages afforded by bilingualism, then they may also extend to reading. We note that although there are a few studies that have found support for a bilingual advantage for reading, these considered bilinguals in aggregate and made comparisons between bilingual and monolingual students on tests of early literacy in young children (Bialystok et al., 2005; Bialystok, Majumder, & Martin, 2003), rather than considering individual differences in a bilingual sample in older students as in this study.

With our individual differences approach to examining how aspects of bilingualism impact reading, we also followed the literature in choosing balance as a variable that may be related to differences in reading outcomes. There is some work in the bilingual advantage literature supporting the construct of balance (i.e., Verreyt et al., 2016), but our study expands upon this by considering reading as the outcome of interest and also by utilizing metrics of balance that integrate L1 and L2 proficiency levels. The latter is particularly important in at-risk populations such as the struggling reader ELs of the current study, because benefits associated with balance may be impacted by the overall level(s) of language proficiency.

Studies relating balance to reading are more emergent and have only been conducted with younger bilingual samples (Grimm et al., 2019; Lonigan et al., 2018); a theme in these studies is to utilize methods that integrate both balance and proficiency through latent profile analysis. We expanded upon this work by considering the role of balance and language proficiency in a similar way in an older sample of children with lower levels of language proficiency and reading achievement than prior studies (and also by including a self-report measure of language, discussed further below). While Lonigan et al. (2018) did not find support for the role of balance, as they found that only English oral language predicted English preliteracy skills, Grimm et al. (2019) found that their balanced group outperformed unbalanced groups on reading comprehension in a sample of second-graders. However, the balanced group in the Grimm et al. (2019) study also demonstrated the highest overall performance across English and Spanish language tests, which begs the question of whether it was balance, or simply high English language proficiency, that drove their results. Since we considered both language proficiency levels and metrics that integrate proficiency with balance and found that only English proficiency remained significant in these models, our findings suggest that balance (at least in the ways operationalized here) did not play a significant role.

### 6.3. The incremental role of language usage for reading outcomes

We also sought to position a self-report measure of language alongside objective measures in the prediction of reading outcomes in order to better understand how contextual information such as usage of L1 and L2 across contexts may influence L2 reading. We were specifically interested in how a higher level of balanced usage, versus dominant English or Spanish usage, may impact reading outcomes. Importantly, we are not aware of any prior studies that have examined such a relationship. We generally did not find support for self-reported language usage to predict reading outcomes. For reading comprehension, though, language usage did have a positive impact on performance. However, it was English usage (relative to balanced usage) that showed this effect, which is conceptually consistent with the more general finding that L2 skills showed stronger effects than those of L1 or of balance. We are somewhat cautious in our conclusions given that the psychometric properties of this measure were less than ideal and so advocate for further work in this area. For example, self-ratings of language usage could be examined in a more detailed fashion, perhaps by adapting measures that have been developed and validated for similar purposes with bilingual adults (Anderson, Mak, Chahi, & Bialystok, 2018).

### 6.4. Limitations and future directions

Current findings should be considered in light of a few limitations. First, as noted above, examination of the roles of L1 and L2 proficiency as well as metrics that integrate balance and proficiency in L2 reading may have yielded different results had we considered non-struggling readers and/or had students with strong L1 and L2 skills been represented within our sample. It is possible that a higher level of language proficiency, especially in L1, as well as stronger foundational L2 reading skills (i.e., decoding), may have allowed L1 skills to bolster L2 reading outcomes, as shown in prior work (Nakamoto et al. 2008;Proctor et al., 2006). Testing this directly in a future study (e.g., evaluating moderation hypotheses that the effects of L1, L2, and their balance could operate differently at different levels of reading and/or language skill) could be useful. Nonetheless, the current findings are important, novel, and informative given how understudied is the population represented by the current sample.

It is also relevant to note that although we did not find support for the role of L1 skills in L2 reading, this does not mean that L1 abilities are not valuable. For instance, Kittle et al. (2024), in their review of reviews, indicate that several aspects of L1 (language and reading) can be leveraged to improve English language and reading outcomes. To this point, the present study did not employ additional measures of the Spanish language (e.g., phonological awareness), or of Spanish reading achievement, to evaluate contrasting hypotheses about the roles of L1 and L2 processes as well as metrics of balance in L1 reading achievement. In addition, our cross-sectional study design did not allow us to consider the role of L1 skills in bolstering the development of L2 skills over time, or in other functional domains. For example, there is some evidence to suggest that L1 skills may contribute to math achievement in L2 (de Araujo, Roberts, Willey, & Zahner, 2018; Peng et al., 2020). The focus of this study does however add important information about proficiency and balance for English reading outcomes in ELs who are struggling readers.

Although reading was our primary target of interest because there is currently a stronger evidence base from which to inform intervention for reading relative to domain general skills, future studies could benefit from also including EF tasks in their designs in order to understand whether any EF advantages are related to balance in this population and how EF may or may not serve as a mechanism through which language-related variables impact reading performance. For example, the extent of switching between languages has been implicated in EF (Crivello et al., 2016; Hernandez, Dapretto, Mazziotta, & Bookheimer, 2001), although results are inconsistent (e.g., Jylkkä et al., 2017). Switching can be measured directly through experimental tasks (Crivello et al., 2016), through functional neuroimaging (Hernandez et al., 2001), and/or via self-report measures of language switching (Anderson et al., 2018). Similarly, Gullifer and Titone (2019) recently proposed the concept of language entropy, which takes into account the extent to which individuals are compartmentalized versus integrated with regard to their L1 and L2 usage within a single context.

As noted, considering the extent to which Spanish skills potentially bolster English language and reading skills as students are exposed to vocabulary that is increasingly Latin-based (e.g., at the later middle school and high school levels) may also help elucidate the current findings. Thus, future work evaluating the roles of L1 and L2 skills in reading could specifically evaluate the word etymology of the reading outcomes in order to examine whether language skills are differentially predictive of Germanic versus Latin-based words.

Finally, our models, which focused on language predictors in total, accounted for a moderate proportion of variance in reading outcomes (29% to 36%). Given the nature of our sample, restriction of range might have limited correlations between our language variables and reading outcomes; however, there was still substantial heterogeneity within the sample (see Table 1). Consideration of cultural, socioemotional and contextual factors may also have increased predictive power. For instance, the Componential Model of Reading (Aaron, Joshi, Gooden, & Bentum, 2008) includes domains for cognition, psychological processes and ecological factors. Psychological variables, including reading motivation and acculturation, may be particularly important to consider in samples of bilingual children, as suggested by a recent study which sought to validate this model in a bilingual context (Li, Koh, Geva, Joshi, & Chen, 2020).

### 6.5. Summary

The current study utilized a nuanced, strengths-based approach to examine how aspects of the bilingual experience impact reading by integrating theoretical frameworks from the bilingual and reading literatures in order to investigate the roles of language proficiency, balance and self-reported language usage in word reading, fluency and comprehension. Results highlight the importance of L2 skills in L2 reading outcomes, with limited findings for the roles of L1 skills or balanced bilingualism in reading. Findings for the self-report measure were also limited (to English usage for reading comprehension) and could be considered in different ways for future work. Overall, this work sheds light on the ways in which variability in language skills impacts an important academic domain in ELs who are struggling readers, with the goal of using this enhanced understanding to inform future intervention efforts.
